# Therapeutic Strategies by Regulating Interleukin Family to Suppress Inflammation in Hypertrophic Scar and Keloid

**DOI:** 10.3389/fphar.2021.667763

**Published:** 2021-04-20

**Authors:** Dan Zhang, Bo Li, Muxin Zhao

**Affiliations:** Department of Plastic and Cosmetic Surgery, The Second Hospital of Dalian Medical University, Dalian, China

**Keywords:** keloid, hypertrophic scar, interleukin, inflammation, therapy

## Abstract

Hypertrophic scar (HS) and keloid are fibroproliferative disorders (FPDs) of the skin due to aberrant wound healing, which cause disfigured appearance, discomfort, dysfunction, psychological stress, and patient frustration. The unclear pathogenesis behind HS and keloid is partially responsible for the clinical treatment stagnancy. However, there are now increasing evidences suggesting that inflammation is the initiator of HS and keloid formation. Interleukins are known to participate in inflammatory and immune responses, and play a critical role in wound healing and scar formation. In this review, we summarize the function of related interleukins, and focus on their potentials as the therapeutic target for the treatment of HS and keloid.

## Introduction

Hypertrophic scar (HS) and keloid are a kind of FDPs, which mainly manifested by fibroblast proliferation and excessive deposition of extracellular matrix (ECM) (Jiang and Rinkevich, 2020). HS is mostly caused by surgery and severe burns, while keloid may result from minor skin damage, such as ear holes, vaccination, acne, etc (Lee and Jang, 2018). Chronic infections and repeated injuries may enhance scar formation and tissue fibrosis (Vinaik et al., 2020). In morphology, they both exhibit hyperplasia, bulge and redness, but unrestrained growth is a specific feature in keloid, which usually invades beyond the margins of the original wound. A majority of patients have to suffer obvious itchiness and pain for a long time (S. S. Lee et al., 2004). Sometimes it not only affects esthetics but also produces dysfunction. At present, there is still lack of efficient curative treatments. Conventional surgery, radiotherapy and hormonal therapy are difficult to achieve complete cure, especially in keloid due to high recurrence rates ( Arno et al., 2014).

Although studies on scars are numerous, the specific pathogenesis mechanism remains unclear in HS and keloid. However, accumulating evidences exist on close link among the inflammation, immune, and pathological scar (Xue et al., 2000; G. Zhu et al., 2007). In the past, keloid and HS were divided into distinct diseases because of the differences in clinical manifestations and pathology (Arno et al., 2014). However, recently, some studies indicate that the difference between them iwas only result of the different duration and intensity of inflammation. Keloid could be defined as pathological scar with severe inflammation, and HS defined as scar with weakly inflammation (Berman et al., 2017). Thus, inflammation as an initial factor will trigger the subsequent immune response cascade, which leads to scar formation. After cutaneous injury, inflammation responses first occur at the site of injury. During the early phase, neutrophil infiltration is the main feature, and the late stage is characterized by monocytes composed of macrophages and lymphocytes (Shimizu et al., 2006). These cells secrete a large number of inflammatory factors such as interleukins, interferons and growth factors, among which interleukins play a prominent role in the initiation of inflammation and subsequent proliferation and remodeling (Abdou et al., 2014). Interleukins act as a major inflammatory factor, potentially regulating fibroblasts recruitment, proliferaton, differentiation, apoptosis and production of ECM. We have observed that the expression of interleukins in scar is different from that of in normal skin (NS). By regulating of interleukin and its related pathways, the phenotype of fibroblasts enhanced or weakened, which suggested that interleukin may serve a significant role in scar. Taken together, we summarize the specific types and changes of interleukins in HS and keloid, and generalize the possible role of regulating the expression and/or secretion of a certain interleukin for abnormal scar prevention.

## IL-6

Interleukin-6 (IL-6) is a proinflammatory factor and a potent immunomodulatory agent (Quong et al., 2017). IL-6 played an important role in chemotaxis and inflammatory cell activation, which was expressed immediately after cutaneous injury and substained over a period of time (Murao et al., 2014). IL-6 could trigger the transition from acute inflammation to chronic inflammation by enhancing monocyte recruitment (Kaplanski et al., 2003). A number of studies had shown that the single nucleotide polymorphism of IL-6 gene affected serum levels. IL-6-572 GG genotype was associated with increasing the risk of keloid in Egyptian (Abdu Allah et al., 2019), Southeastern Chinese (X. J. Zhu et al., 2017) and Japanese (Tosa et al., 2016). Both IL-6 and IL-8 decreased significantly in early fetal fibroblasts (Namazi et al., 2011), which may help to produce scarless healing. IL-6 secretion was higher in HS and keloid, and the mRNA and protein levels of IL-6R α and IL-6R β (gp130) and its downstream targets LAK1, STAT3, RAF1 and ELK1 were up regulated (Ghazizadeh et al., 2007).

### Direct Effect of Targeting IL-6

IL-6 may be an effective target in treating HS and keloid. When IL-6 peptide was added into human normal fibroblasts (NFs), the expression and synthesis of collagen were revealed a dose-dependent increase (Ghazizadeh et al., 2007). If monoclonal anti-IL-6 or anti-IL6Ra antibody were added, the progress would be prevented. Ray et al. (Ray et al., 2013) also demonstrated that IL-6 increased the production of ECM and cellular proliferation mediated by STAT3 pathway in HS. Counter regulating the overexpression of IL-6 may be a key trigger factor to inhibit the prolongation of inflammatory phase in keloid wound healing (Euler et al., 2019).

### Regulation of IL-6 Expression and/or Secretion

**FIGURE 1:**
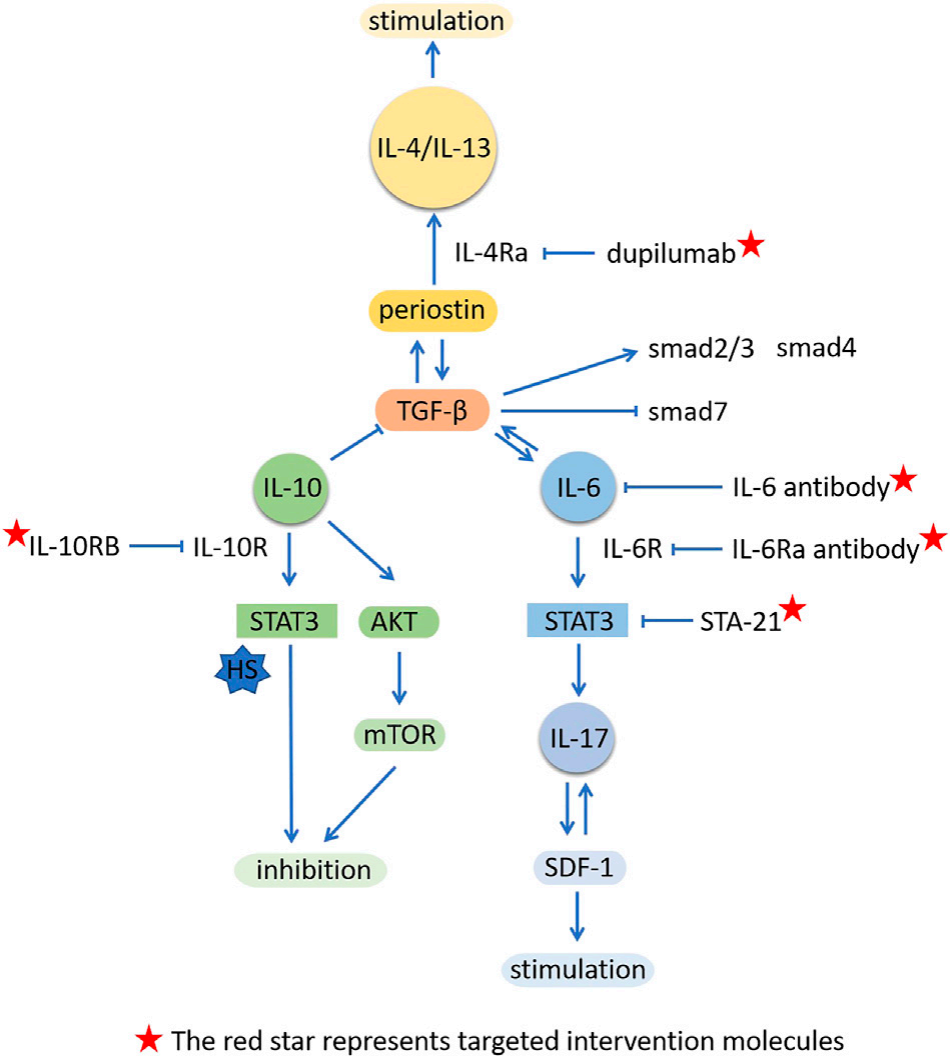
Modulating interleukins or their related signal molecules in scar formation.

In addition, modulating the IL-6 signaling pathway may also affect the wound healing and scar formation (Figure 1). Currently, several treatments for HS and keloid are known to regulating the expression and/or secretion of IL-6 (Table 1). We here summarized these treatments as follows.

**TABLE 1:** Treatments regulating IL-6 in HS and keloid.

Treatment	Disease	Pathway	Effect	References
ARB/ACEI	HS/keloid	IL-6/TGF-β and AP-1/TGF-β	IL-6,TNF-α and TGF-β↓	Hedayatyanfard et al. (2020)
TA	HS/keloid		IL-6 and VEGF↓	Johnson et al. (2020)
BTA	HS/keloid	JNK	TGF-β, IL-6, CTGF and side effects↓	L. Chen et al. (2019)
Verapamil	HS/keloid		IL-6 and VEGF↓	Foubert et al. (2017), Giugliano et al. (2003)
Pirfenidone	HS		IL-6, IL-2 and neutrophil infiltration↓	Medina et al. (2019)
Sulforaphane	keloid	STAT3/Smad3	IL-6↓	Kawarazaki et al. (2017)
PTXL	keloid	Akt/GSK3β	TNF-α, IL-6 and TGF-β↓	M. Wang M. et al. (2019)
Emodin	HS	PI3K/Akt	TNF-α, IL-6 and MCP-1↓	C. Liu (2015)
Curcumin	HS/keloid		IL-1, IL-6 and IL-8↓	Jia et al. (2014)
Naringin	HS		TNF-α, IL-1β, IL-6 and TGF-β1↓	Shan et al. (2017)
Ginseng	HS		NF-κB, TGF-β1, IL-6↓	Pazyar et al. (2012)
Ginsenoside Rg3	HS	NF-κB/IκB	IL-1β, IL-6, and TNF-α↓	Ma et al. (2020)
GNA	HS		TGF-β1, CTGF, IL-1β, IL-6, TNF-α↓ IL-10↑	Jun et al. (2021)
Lapadan	HS		IL-6 and PAI-1↓	Matsui et al. (2011)
Stem cells	HS/keloid		Inflammation and fibrosis↓ but UCMSCs are controversial (IL-6↓/↑)	Fong et al. (2014), J. Liu et al. (2018), Fang et al. (2016), Jiao et al. (2017), Arno et al. (2014a)
HBOT	keloid		Inflammatory factor↓ (IL-6↓/-)	Song et al. (2018), Y. Hao et al. (2020)
IFN-γ	keloid	JAK/STAT3 and Jak1/STAT1	IL-6 and col1 synthesis↓	Euler et al. (2019)
TSG-6	HS/keloid		IL-1β, IL-6 and TNF–α↓	H. Wang H. et al. (2015)
SVF-gel/cells	HS		IL-6 and MCP-1↓	J. Wang J. et al. (2019)
PLM	HS		IL-6 and TNF-α level in both serum and tissuse↓	Demircan et al. (2021)
UVA	keloid	p38/NF-κB1	IL-6 and IL-8↑	Niu et al. (2020)

#### Synthetic Drugs

Initial results with angiotensin receptor blocker (ARB) and angiotensin converting enzyme inhibitor (ACEI) in the treatment of keloid and HS were encouraging (Kilmister et al., 2019). Renin angiotensin aldosterone system (RAS) components which are expressed in various cells of skin and act independently of plasma RAS, play an important role in wound healing and scar formation. By acting on AT1 receptor (Mulrow. 1999), angiotensin II promoted fibrosis, induced migration and proliferation of keratinocytes and fibroblasts, and increased collagen production through IL-6/TGF-β and AP-1/TGF-β pathways (Hedayatyanfard et al., 2020). The activation of AT2 receptor inhibited the above-mentioned process by blocking the expression of IL-6, TNF-α and TGF-β, and played an anti-inflammatory role (Hedayatyanfard et al., 2020). Triamcinolone acetonide (TA) has been routinely used as a treatment for keloid. It was a corticosteroid hormone that could reduce the expression of IL-6 and vascular endothelial growth factor (VEGF) (Johnson et al., 2020). Botulinum toxin A (BTA) is effective on keloid and HS (R. Hao et al., 2018). In the keloid model of athymus mice, BTA reduced inflammatory infiltration and collagen tissue, comparing with the gold standard triamcinolone acetonide, and the former showed less side effects (Fanous et al., 2019). Chen (L. Chen et al., 2019) showed that BTA could reduce the proliferation and migration of fibroblasts through JNK pathway activation, and inhibit the protein expression levels of TGF-β, IL-6 and connective tissue growth factor (CTGF). In contrast, in a culture with dermal fibroblasts and microvascular endothelial cells, the above phenomenon was not observed (Haubner et al., 2012). Verapamil is also a controversial drug for scars. It was a calcium channel blocker (W. Wang et al., 2016), which could inhibit the growth of central keloid fibroblasts (KFs) , induce apoptosis, reduce the production of IL-6 and VEGF and the synthesis of ECM (Giugliano et al., 2003; Foubert et al., 2017). This treatment was considered safe (Verhiel et al., 2015), but there was still no consensus on the therapeutic value (Danielsen et al., 2016; Abedini et al., 2018). Due to some methodological defects, it needs larger samples and better models to verify. Pirfenidone, as an FDA approved antifibrotic drug, regulated wound inflammation by reducing the expression of inflammatory factors such as IL-6, IL-2 and neutrophil infiltration in deep burn model of mice, which reduced the formation of scar (Medina et al., 2019).

#### Natural Extraction Medicine

In addition to conventional drugs, some ingredients from natural plants also play a role in the treatment of HS and keloid. Sulforaphane, an isothiocyanate extracted from cruciferous vegetables (such as broccoli sprouts), down-regulated the expression of IL-6 in KFs by inhibiting NF-κB, and also inhibited STAT3 and smad3 signal transduction pathways (Kawarazaki et al., 2017). Paclitaxel (PTX) was an effective chemotherapeutic drug, which has been reported to have anti-fibrosis effect (C. Wang et al., 2013). Its modified form (PTXL) could inhibit the growth and invasion of keloid, and showed better effect. At the same time, PTXL also reduced the production of fibrosis cytokines including TNF-α, IL-6 and TGF-β by inhibiting Akt/GSK3β signaling pathway, thus preventing the fibrosis process of keloid (M. Wang M. et al., 2019). Emodin was the main component of rhubarb, which had the same inhibitory effect, but mainly targeted PI3K/Akt pathway (C. Liu, 2015). Curcumin was a yellow organic compound purified from the rhizome of curcuma longa, which had good anti-inflammatory properties (Menon and sudheer, 2007). It inhibited the proliferation, contraction and ECM production in primary KFs (Phan et al., 2003). Fourthmore, it suppressed the formation of HS by reducing the secretion of IL-1, IL-6 and IL-8 (Jia et al., 2014), which the same as naringin (Shan et al., 2017). IL-6 level was also influenced by other natural extracts such as Ginseng (Pazyar et al., 2012), Ginsenoside Rg3 (Ma et al., 2020), Gambogenic acid (GNA) (Jun et al., 2021) and Lapadan (Matsui et al., 2011), which reduced the rates of hypertrophic scar formation.

#### Stem Cells

Stem cells also have important implications by secreting a large number of nutritional factors and anti-fibrosis factors for fibrosis diseases. Studies have shown that umbilical cord mesenchymal stem cells (UCMSCs) (Fong et al., 2014), adipose stem cells (ASCs) (J. Liu et al., 2018), bone marrow stem cells (BMSCs) (Fang et al., 2016), fetal dermal stem cells (FDSCs) (Jiao et al., 2017) and their corresponding conditioned media (CM) have a good inhibitory effect on scar fibroblasts.

After 5 days of co culture of ASCs with hypertrophic scar fibrolasts (HSFs), fibrosis factors such as IL-6, IL-8, FN, and TGF-β1 were significantly decreased, while the protective factors decorin (DCN) and the ratio of matrix metalloprotinase-1 (MMP-1)/tissue inhibitor of metalloproteinase-1 (TIMP-1) were significantly increased (Deng et al., 2018). Moreover, ASCs reversed the occurrence of fibrosis and inflammation induced by TGF-β (Deng et al., 2018). ASC-derived CM inhibited the secretion of IL-6, IL-8, α-SMA and proliferation and migration of KFs (J. Liu et al., 2018). In the red Duroc (RD) pig porcine model, delivery of autologous adipose derived regenerated cells (ADRC) immediately resulted in an up-regulation of IL-6 at 2 weeks after injury (wound healing stage) and down-regulation at 2 months after treatment (early scar formation stage) (Foubert et al., 2017). The early inflammatory reaction was beneficial to the wound healing, and the later inflammatory reaction was inhibited which reduced the probability of HS formation. It showed better wound healing, less pigmentation and stiffness, more normal collagen tissue and lower vascular density (Foubert et al., 2017). A similar view was also confirmed in UCMSCs (Bonnardeaux and McCuaig, 2020). However, reports on UCMSCs are not consistent. Another study found that the supernatant from UCMSCs promoted fibrosis phenotype as a whole, and increased the production of inflammatory factors such as IL-6, IL-8 and TGF-β (Arno et al., 2014a). The inconsistency may be due to the varied sources of UCMSCs. Stem cells from different locations have different secretory groups, resulting in differential expression.

#### Hyperbaric Oxygen

High fibroblasts proliferation in keloids led to hypoxia condition (Jusman et al., 2019), which induced HIF-1α production (Z. Zhang et al., 2014). HIF-1α expression in fibroblasts stimulated TLR/MyD88/NF-κB signaling pathway and promoted the expression of some inflammatory factors such as IL-6 (Y. Hao et al., 2020). According to the mechanism of hypoxia-induced inflammation, several clinical experiments confirmed that adjuvant hyperbaric oxygen treatment (HBOT) reduced the inflammatory reaction and recurrence rate by regulating the oxygen level. In Song’s study (Song et al., 2018), 240 patients with keloid were recruited and randomly divided into two groups, Group O receiving HBOT after surgical resection and radiotherapy and Group K as control. After HBOT, both the infiltration of inflammatory cells and the expression of inflammatory factors such as IL-6, HIF-1 α, TNF-α, NF-κ B and VEGF obviously decreased. However, in another experiment (Y. Hao et al., 2020), no significant difference was observed in the expression of IL-6, IL-8, or IL-10. In this experiment, there were only 10 cases in each group, and HBOT was used before operation. Different treatment combinations may have different effects on the secretion of cytokines.

#### Other Methods

The production of IFN-γ in the serum of normal patients was higher than keloid persons (Mccauley et al., 1992), which may cause keloid formation by increasing IL-6 secretion. IFN-γ regulated the secretion of IL-6 (Xue et al., 2000) by means of stimulating MHCII and CD40 expression (Yellin et al., 1995) and associate with JAK/STAT pathway (Euler et al., 2019). The combined use of IFN-γ/TA is better than any single use (Mccauley et al., 1992). TSG-6 exhibited anti-inflammatory activity (R. H. Lee et al., 2009). When TSG-6 was intradermally injection into ear wounds, it resulted in lower secretion levels of IL-1 β, IL-6 and TNF–α (H. Wang et al., 2015). Moreover, the expression of TSG-6 protein in keloid was decreased (Tan et al., 2011). It is likely that exogenous TSG-6 may significantly diminish the development of keloid. Injection of Stromal vascular fraction (SVF)-gel or SVF cells reduced the macrophages infiltration in dermal layer, and decreased mRNA expression of IL-6 and MCP-1, so that the level of myofibroblasts and collagen deposition were reduced (J. Wang et al., 2019). SVF gel also restored subcutaneous adipose tissue and made HS appear soft and unobvious (J. Wang J. et al., 2019). Silver containing hydraulic fiber (HFAg) and polylactic acid membrane (PLM) are two different burn dressings. Compared with the HFAg, IL-6 and TNF-α levels decreased in early days in both serum and tissue samples to reach normal ranges by PLM, which would prevent the development of HS (Demircan et al., 2021). Reports on UVA are contriversial for keloid. In general, UVA had a good effect on KFs, but it activated p38/NF-κB pathway, which increased the release of IL-6 and IL-8 and the overall inflammatory response (Niu et al., 2020). Therefore, the simple cellular phenotype can not fully explain the controversy, and *in vivo* experiments are needed to verify the effect of UVA.

## IL-10

The expression of IL-10 was decreased in keloid (J. H. Shi et al., 2013; Z. Chen et al., 2018) and HS (Yang et al., 2018). Intrinsic lack of IL-10 may result in continued amplification of the inflammatory cytokine cascade, continued stimulation of fibroblasts, and abnormal collagen deposition (Liechty et al., 2000). Peranteau et al. (Peranteau et al., 2008) showed that overexpression of IL-10 decreased inflammation and created an environment for wound sites in the adult to more closely resemble the profile seen in the embryo. IL-10 inhibited the secretion of IL-6 and IL-8, which induced inflammatory cascade reaction, promoted fibroblasts proliferation and collagen synthesis (Mccauley et al., 1992; Liechty et al., 2000M. Zhang et al., 2016). According to these results, the use of recombinant human (RH) IL-10 may be a potential treatment for keloid. And better results may be achieved through some new assembly methods and means.

### Direct Effect of Targeting IL-10

IL-10 itself could directly inhibit the growth of KFs, but it had no effect on normal scar fibroblasts (NSFs), which was the same as Ji's report (J. Shi et al., 2014), but this was slightly different from that reported by Moroguchi (Moroguchi et al., 2004), who observed that IL-10 inhibited the proliferation of fibroblasts induced by TNF-α (Shi et al., 2019). This may be caused by different cell lines and concentrations of IL-10. Shi et al. (J. H. Shi et al., 2013) found that the injection of IL-10 improved the morphology of scar, inhibited the contracture of wound, narrowed the edge of wound, and relieved the deposition of collagen (col1 and col3) in regenerated tissue. Lentivirus-mediated overexpression of IL-10 reduced the inflammatory response to injury after 3°days, showing a favorable environment for wound healing (Peranteau et al., 2008). On the contrary, if the IL-10 gene was knocked out, the injury sites would present an increased inflammatory response and excessive collagen deposition (da Silva et al., 2017). IL-10 RB, a function-blocking antibody against the IL-10 receptor, blocked the IL-10-mediated mitigation of fibrosis in HSFs (J. Shi et al., 2014). And it has also been verified in clinical human experiments. The experiment of Kieran et al. (Kieran et al., 2013) well illustrated that the appearance and histology of scar treated with exogenous RH IL-10 were improved. Denervated wounds could lead to abnormal wound healing and caused hypertrophic scars. In addition, when IL-10 was added to the injury model of CD1 mice, the patterns of reinnervation and revascularization were improved and the collagen tissue of the dermis was closer to the normal skin healing than the control (Henderson et al., 2011).

### Regulation of IL-10 Related Pathways

After treatment with IL-10, the expression of col1 and col3 in KFs was significantly reduced, and the levels of TGF-β, smad2/3 and Smad4 were down-regulated, while the level of smad7 was up-regulated, suggesting that IL-10 inhibited the formation of keloid by inhibiting the classic TGF-β/Smad signal pathway of fibrosis (Shi et al., 2019). Lipopolysaccharide (LPS) could induce NFs into HFs and participate in the formation of HS by TLR4/NF-κB pathway (J. Shi et al., 2021), and promote the production of inflammatory molecules (J. Wang et al., 2011). The addition of IL-10 inhibited the inflammation and fibroblast filled collagen lattice (FPCL) contracture induced by LPS, which was associated with regulating the IL-10R/STAT3 axis of TLR4/NF-κ B pathway in skin fibroblasts (J. Shi et al., 2021). In the rabbit ear hypertrophic scar model, BMSCs modified with IL-10 inhibited the expression of TNF-α, IL-6 and IL-1 β mRNA through JNK/NF-κ B pathway, which significantly reduced the wound healing time, scar area and height (Xie et al., 2020). IL-10 protected HSFs from fibrosis by activating Akt and STAT3 signal transduction pathways to reduce collagen production (J. Shi et al., 2014). Moreover, skin autophagic capability is associated with HS. IL-10 also inhibited starvation-induced autophagy, which was mediated by the cross talk IL10-IL10R-STAT3 and IL10-AKT-mTOR pathways (J. Shi et al., 2016) (Figure 1).

### A New Method of Targeting IL-10

In order to improve the function of IL-10 and reduce the side effects, new methods have been developed which enhance the function of IL-10 through some corresponding combination and packaging means. Park et al. (Park et al., 2019) developed a new delivery platform coacervates (COA), which had features of high biocompatibility, easy preparation and better protection of growth factors including TGF- β3 and IL-10 (Lichtman et al., 2016). Both TGF-β3 and IL-10 had anti-fibrosis effect in physiological wound healing process. Shi et al. (J. Shi et al., 2015) designed a novel hybrid protein RHIL10-RGD, which was fused and expressed in *E. coli* BL21 (DE3). Treatment of HSFs with RH IL10 RGD was similar to that of RH IL10 in anti-fibrosis, but the former could specifically reduce angiogenesis. In addition, the Orf viru encoded vascular endothelial growth factor (VEGF)-E and interleukin-10 (ovil-10), which synergistically enhanced skin repair, and acted in a complimentary fashion to improve scar quality (Wise et al., 2020).

## IL-1

IL-1, also known as lymphocyte stimulating factor, has two forms: IL-1α and IL-1β (Gabay et al., 2010), which are mainly produced by monocyte macrophages. IL-1 played an important role in the early stage of scar formation (Jfri et al., 2019). Although impaired production of keratinocyte-derived growth factors, such as IL-1α, lead to a decrease in the catabolism of the dermal (Niessen et al., 2001), the role in scar formation of IL-1α remains controversial. However, IL-1β occupied a more important position in keloid (Vinaik et al., 2020; Le et al., 2021) and HS (Krotzsch-Gomez, 1998). IL-1 β was overexpressed in HS at post transcriptional level, and it participated in ECM remodeling with TNF-α to maintain fibrosis phenotype (Salgado et al., 2012). The level of IL-1β predicted the formation of HS (Kwan et al., 2016).

### Exogenous IL-1 β Promotes Scar Formation

IL-1 β enhanced the signal of fibrosis in the late stage of wound healing, such as increased the expression of MCP-1, while MCP-1 and IL-6 synergistically promoted inflammation (Kawarazaki et al., 2017). The addition of IL-1β to NFs and HSFs could lead to oxidative stress and regulate cell apoptosis, such as the increased of heat shock transcription factor-1,IL-6 and HSP-70 and the decreased of NF-κB, GADD45-α, p53 and p53 binding proteins. The oxidative stress and heat stress proteins induced by IL-1 β are important mediators of abnormal scar formation after severe burns (Barrow and Dasu, 2005) IL-1β also decreased the endogenous Prostaglandin E2(PEG2)secretion in KFs, which inhibited cell migration and contraction, and down regulated collagen synthesis (Sandulache et al., 2007). These factors may jointly promote scar formation.

### Blocking IL-1 β Inhibits Scar Formation

The expression and activity of chymase in keloid tissue were increased, which promoted the formation of keloid through IL-1β, col1 and TGF-β1 (R. Wang R. et al., 2015). Blocking chymase pathway may be an effective method to improve keloid. Inflammatory body is the main regulator of inflammation and metabolic response. The activation of NLRP3 facilitated cleaved caspase-1 processing and promoting the release of IL-1β and IL-18, and increased the inflammatory response in keloid (Vinaik et al., 2020). Moreover, the use of NLRP3 inhibitor MCC950 reduced the expression of IL-1β (Ogawa, 2017; Perera et al., 2018), and inhibiting the activation of IL-1 β in inflammatory body may be a method to inhibit keloid. The expression level of IL- 1 β in epidermis was directly related to the degree of skin fibrosis. In the rabbit ear hypertrophic scar model, Corrie l et al. (Gallant-Behm and Mustoe, 2010) confirmed that occlusion with silicone gel increased the hydration state of epidermis in a dose-dependent manner, and inhibited fibrosis and alleviated HS (Sandulache et al., 2007) by significantly reducing the epidermal expression of profibrotic cytokine IL-1β. Tranilast inhibited fibroblasts proliferation through lower the production of IL-1β by macrophages and other inflammatory cells, thus lessen keloid (Suzawa et al., 1992). Beyond that,Collagen-polyvinylpyrrolidone also changed the inflammatory process of HS by reducing the expression of proinflammatory cytokines IL-1 β and TNF-α (Krotzsch-Gomez, 1998).

Interleukin-1 receptor antagonist (IL-1RA) is a member of the IL-1 gene family, which binds to IL-1R and specific blocks the activity of IL-1 (Gabay et al., 2010). In the New Zealand rabbit model, administration of IL-1RA effectively reduced skin fibrosis, and the expression of downstream signal C-FOS was blocked (Gallant-Behm et al., 2011). In addition, the expression of IL-1RA was increased in the treatment of keloid by HBOT in the clinical experiment of Hao (Y. Hao et al., 2020), which reduced the level of inflammation and played a better curative effect. Therefore, suppressing the expression of IL-1 β attenuates scar formation.

## Other Interleukins

### IL-4/IL-13

There was evidence that the expression of interleukin-4 (IL-4) (Tredget et al., 2006; Yang et al., 2018), interleukin-13 (IL-13) (Li et al., 2015) and their receptors (Diaz et al., 2020) were increased in pathological scars. IL-13 and IL-4 up-regulated the expression of collagen related genes, inhibited the degradation of collagen induced by MMP-1 and MMP-3, and promoted collagen deposition (Oriente et al., 2000). Compared with wild-type mice, the scarring formation in T-cell-deficient mice was reduced by nearly nine fold, which may be closely related to the decreased expression of T-cell-dependent Th2 cytokines (IL-4 and IL-13) and chemokines (MCP-1) (Wong et al., 2011).

Maeda et al. demonstrated that IL-4 and IL-13 induced the expression and secretion of periostin, which in turn to induce RhoA/ROCK pathway to mediate the TGF-β1 expression (Maeda et al., 2019). TGF-β1was recognized as one of the important factors in keloid formation (Berman et al., 2017), which in turn acted as periostin, forming a positive feedback loop (Maeda et al., 2019). Dupilumab is a completely humanized monoclonal antibody against IL-4Rα. Unexpectedly, it had inhibitory effect on keloid associated with atopic dermatitis. Based on this, the same results were obtained in the treatment of three cases of chronic keloid without atopic dermatitis (Diaz et al., 2020).

### IL-17

Interleukin-17 (IL-17) is an inflammatory factor produced by CD4^+^T cells (Chang, 2019), which can promote the activation of T cells and stimulate the production of IL-6, IL-8, and so on, leading the inflammation. IL-17 is considered as a marker of Th17 cell subsets (Miossec and Kolls, 2012). The infiltration of Th17 cells in keloid was increased (Le et al., 2021), and IL-17 was up-regulated in keloid and HS (J. Zhang et al., 2018). IL-17 promoted the expression of α-SMA and Col1. Compared with normal skin dervied precursor cells, keloid derived precursor cells (KPCS) expressed higher IL-17R. When altered keloid niche (mainly inflammation), which was mediated by IL-6/IL-17 axis through autocrine or paracrine, and showed uncontrolled self-renewal and increased proliferation (Q. Zhang et al., 2009). Besides, IL-17 promoted the expression of stromal cell-derived factor 1 (SDF-1) and increased the recruitment of Th17 cells from the circulatory system. This positive feedback loop may lead to excessive infiltration of T cells and chronic inflammation of keloid. Sta-21 reduced the expression of SDF-1 in KFs by inhibiting STAT3 pathway and break the feedback loop (Le et al., 2021). Moreover, IL-17 stimulated mice showed increased fibrosis, which induced macrophage specific subtype infiltration through MCP-dependent mechanism, resulting in delayed wound healing and increased inflammation (J. Zhang et al., 2018).

### IL-18

Interleukin-18 (IL-18) is a member of the IL-1 family (Yasuda et al., 2019). It has the opposite effect to scar formation in keloid and HS. IL-18 in HS tissue and fibroblasts decreased. When the RH IL-18 was injected into the HS model of rabbit ears, and the scar was improved. It was proved that IL-18 inhibited the proliferation and promoted the apoptosis of HSFs by enhancing the expression of FasL (Le et al., 2021). However, not only the expression of IL-18 in keloid tissue, but also the receptors of IL-18R α and IL-18R β was increased (Felipo et al., 2012; M. Zhang et al., 2016; Vinaik et al., 2020). When KFs were exposed to IL-18, the synthesis of collagen and ECM components were increased, and the secretion of fibrinolytic cytokines (such as IL-6 and IL-8) was up-regulated (Do et al., 2012). Moreover, when keratinocytes and keloid fibroblasts (KK/KF) were cocultured, IL-18/IL-18BP was seriously unbalanced, which promoted the formation of keloid (Do et al., 2012). It suggests that IL-18 system plays an important role in the pathogenesis of keloid through epithelial mesenchymal interaction.

### IL-37

Interleukin-37 (IL-37) is a relatively new member of the IL-1 family and has been described as an anti-inflammatory mediator in various inflammatory diseases (Cavalli and Dinarello, 2018). In a cross-sectional study, it was found that the level of IL-37 was negatively correlated with the severity of keloid, but had no significant correlation with age, gender, duration of lesions or family history, indicating that the decrease of plasma IL-37 level could be used as an indicator of keloid severity (Khattab and Samir, 2020). Zhao et al. (Zhao et al., 2020) confirmed the above viewpoint, and observed IL-36 expression in keloid was also decreased. Therefore, the recombinant IL-36 and IL-37 have potential as a novel therapeutic approach in pathological scar, which is warranted in the future.

### IL-22

Interleukin-22 (IL-22) is a member of the IL-10 family (Dudakov et al., 2015). In fibroblasts, IL-22 signal was activated and then guided the expression of extracellular matrix genes and differentiation of myofibroblasts. Significantly increased expression of TGF-β, IL-22 and Arg-1 in keloid was found as compared to normal scar tissue (da Cunha Colombo Tiveron et al., 2018).

### IL-24

Interleukin-24 (IL-24) is also a member of the IL-10 family. The mRNA level of IL-24 in KFs was significantly lower than that in normal skin. The formation of keloid may be correlated with the down-regulation of IL-24. Adenovirus-mediated IL-24 selectively inhibited the proliferation and induce apoptosis of KFs, suggesting that IL-24 has great potential in therapy of keloid (Liang et al., 2011).

## Summary

HS and keloid are both clinical challenge to be solved. The mechanism behind them has not been completely elucidated. Accumulating studies have demonstrated that HS and keloid are associated with inflammation, and thus suppression of inflammation may inhibit scar formation. Accordingly, we summarized the interleukin expression in scar and explored the influences of drugs and methods targeting interleukins.

**FIGURE 2:**
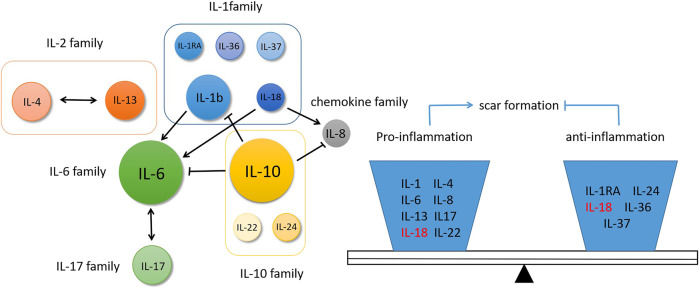
The relationship of interleukins and their functions in scar formation.

In HS and keloid, there is a relatively large number of studies on IL-6, IL-10 and IL-1β. As proinflammatory factors, IL-6 and IL-1β play an important role in promoting scar formation. IL-1β have been shown to stimulate IL-6 production (Ghazizadeh et al., 2007), and they acted additively or synergistically to promote scar formation. IL-6 and IL-17 formed a positive feedback regulation (Q. Zhang et al., 2009), together with classic fibrosis factor TGF-β in the propagation of fibrosis. On the contrary, as an anti-inflammation factor, IL-10 protected the wound healing and made it tend to scarless healing (Seifert and Mrowietz, 2009). At the same time, IL-10 inhibited the pro-inflammatory effect of IL-6 and IL-8 (Liechty et al., 2000) and produced antagonistic effect. Therefore, these interleukin molecules are not isolated, they crosstalk with each other to form a network regulation. During the normal wound healing and scar formation, these inflammation factors exist in equilibrium. Once the balance is broken caused by some stimulating factors, such as infection or trauma, pathological scar is generated. Currently, mote researches demonstrate the upstream or downstream signal molecules of interleukin family as indirect regulators, however it may be more intuitive that directly target certain interleukins. And this regulation has a certain intervention effect on multiple interleukin molecules (Figure 2).

Although some phenotypic and pathway effects have been observed, the crosstalk between interleukins is not fully understood. Some molecules may have two sides and differ strongly functions in varied pathways, which results in potentially contradictory outcomes. For example, in the treatment of keloid with HBOT, song (Song et al., 2018) observed that it could reduce the formation of scar by reducing inflammatory factors such as IL-6 and IL-8, while another experiment (Y. Hao et al., 2020) did not observe the same changes, but the alterations in expression of IL-12p40 and IL-RA were observed. Also, the conditioned medium from UCMCs displayed opposite effects on scar fibroblasts (Arno et al., 2014a; Bonnardeaux and McCuaig, 2020). UVA has a good effect in the overall treatment of keloid, but IL-6 and IL-8 were activated (Niu et al., 2020). The role of IL-18 in HS and keloid was conflicting, which inhibited the former and promoted the latter (Do et al., 2012; Le et al., 2021). These contradictions are worthy of further investigations in the future, and require a larger sample size and better models to validate.

Recently, researchers have also found other interleukins in scar, such as IL-22, which was activated in KFs, involved in EMC synthesis and myofibroblast transformation (da Cunha Colombo Tiveron et al., 2018). Additionally, the serum level of IL-37 was negatively correlated with the severity of keloid lesions, and IL-36, which had not been previously concerned in scars, was also mentioned (Zhao et al., 2020). It is suggested that other interleukins involved in the formation of scar may exist. And there is no intervention on these interleukins. Exogenous application of these molecules may be one of the future directions in the treatment of scar, but the concentration and safety should be paid attention to. In addition, with the development of other technologies such as tissue engineering, we may find better drug combinations and packaging methods, which will drive the development and innovation of interleukin-related interventions.

In a word, interleukins play a significant role in HS and keloid, and targeting some interleukins and suppressing inflammation are important strategies in the treatment of pathological scar.
